# Supplementary figures and images for: A simplified pneumonia severity index (PSI) for clinical outcome prediction in COVID-19
Source: PLoS One. 2024 May 21;19(5):e0303899. doi: 10.1371/journal.pone.0303899 (PMC11108185; doi:10.1371/journal.pone.0303899)

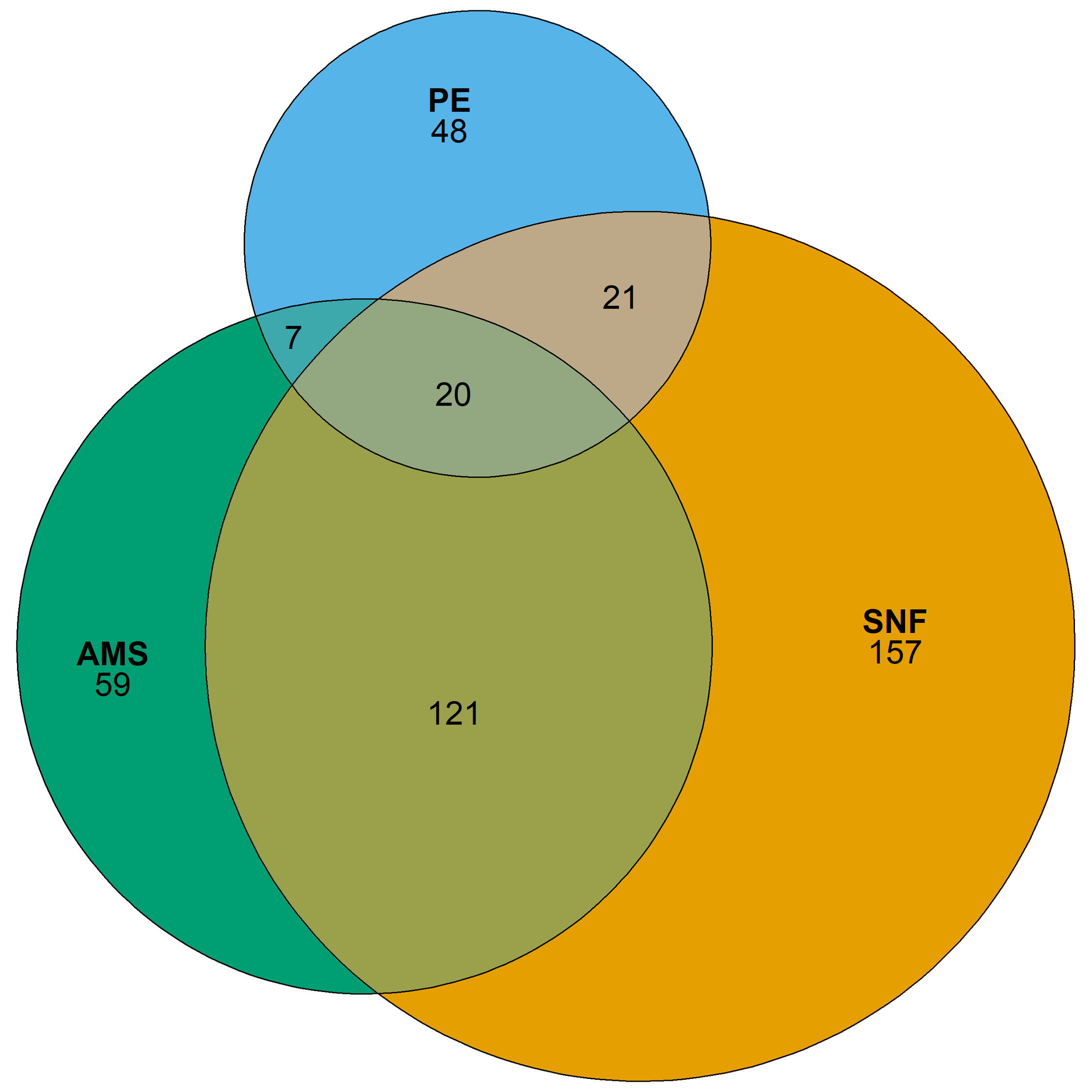

Supplement: S1 Fig — Among 1,138 patients, 433 (38%) patients had at least one of the 3 PSI-20 variables (SNF, skilled nursing facility; AMS, altered mental status; PE, pleural effusion); 705 patients (62%) did not have any of the 3 variables. (TIF) [file pone.0303899.s001.tif]
