# Supplementary material for: A simplified pneumonia severity index (PSI) for clinical outcome prediction in COVID-19
Source: PLoS One. 2024 May 21;19(5):e0303899. doi: 10.1371/journal.pone.0303899 (PMC11108185; doi:10.1371/journal.pone.0303899)
Supplement: S1 Table — (DOCX) [file pone.0303899.s002.docx]

**S1 Table. Points assigned for PSI-20 (Fine et al., 1997) and PSI-17.**

| **Variables** | **Points Assigned*** | |
| --- | --- | --- |
|  | **PSI-20^a^** | **PSI-17^b^** |
| **Demographic factor** |  |  |
| Age | +Age (yr) | +Age (yr) |
| Female | -10 | -10 |
| SNF | +10 | 0 |
| **Coexisting illnesses** |  |  |
| Neoplastic disease | +30 | +30 |
| Liver disease | +20 | +20 |
| Congestive heart failure | +10 | +10 |
| Cerebrovascular disease | +10 | +10 |
| Renal disease | +10 | +10 |
| **Physical-examination findings** |  |  |
| AMS | +20 | 0 |
| Respiratory rate ≥30/min | +20 | +20 |
| Systolic blood pressure < 90 mm Hg | +20 | +20 |
| Temperature <35°C or ≥40°C | +15 | +15 |
| Pulse ≥125/min | +10 | +10 |
| **Laboratory and radiographic findings** |  |  |
| Arterial pH <7.35 | +30 | +30 |
| Blood urea nitrogen ≥30 mg/dl | +20 | +20 |
| (11 mmol/liter) |  |  |
| Sodium <130 mmol/liter | +20 | +20 |
| Glucose ≥250 mg/dl (14 mmol/liter) | +10 | +10 |
| Hematocrit <30% | 10 | 10 |
| Partial pressure of arterial oxygen | 10 | 10 |
| <60 mm Hg |  |  |
| PE | 10 | 0 |

Abbreviations: SNF, skilled nursing facility; AMS, altered mental status; PE, pleural effusion.

^*^ Total point score for a given patient is obtained by summing the patient’s age in years

(age minus 10 for female) and the points for each applicable characteristic.

^a^ Fine et al. (1997)

^b^ Same as PSI-20 without 3 risk factors (AMS, SNF, PE)
