# Supplementary material for: A simplified pneumonia severity index (PSI) for clinical outcome prediction in COVID-19
Source: PLoS One. 2024 May 21;19(5):e0303899. doi: 10.1371/journal.pone.0303899 (PMC11108185; doi:10.1371/journal.pone.0303899)
Supplement: S2 Table — (DOCX) [file pone.0303899.s003.docx]

**S2 Table. Patient demographics and baseline characteristics.**

| **Patients, n** | 1,138 |
| --- | --- |
| ***Demographics*** |  |
| **Age, mean (SD)** | 65.4 (16.5) |
| **Male sex, n (%)** | 630 (55.4) |
| **Race, n (%)** |  |
| White | 569 (50.0) |
| Asian/Pacific Islander | 106 (9.3) |
| Black/African American | 63 (5.5) |
| Hispanic/Latino | 284 (25.0) |
| Other/Unknown | 116 (10.2) |
| **Ethnicity, n (%)** |  |
| Hispanic/Latino | 284 (25.0) |
| Not Hispanic/Latino | 815 (71.6) |
| Other/Unknown | 39 (3.4) |
| **Comorbid Conditions** |  |
| Dementia, n (%) | 324 (28.5) |
| Hypertension, n (%) | 382 (33.6) |
| Cancer, n (%) | 57 (5.0) |
| Liver disease, n (%) | 14 (1.2) |
| Congestive heart failure, n (%) | 77 (6.8) |
| Cerebrovascular disease, n (%) | 86 (7.6) |
| Renal disease, n (%) | 94 (8.3) |
| ***Clinical Features at Admission*** |  |
| BMI, median (IQR) | 28.2 (24.3 – 33.5) |
| PSI, median (IQR) | 77 (55 – 102) |
| Admit from SNF, n (%) | 319 (28.0) |
| Pleural effusion, n (%) | 96 (8.4) |
| **WHO-OSS, n (%)** |  |
| 3 (No O_2_) | 416 (36.6) |
| 4 (low-flow O_2_) | 656 (57.6) |
| 5 (high-flow O_2_) | 48 (4.2) |
| 6 (Mechanical ventilation) | 18 (1.6) |
| **DNR status, n (%)** | 165 (14.5) |
| **AMS, n (%)** | 207 (18.2) |
| **Respiratory rate (per min)** |  |
| median (IQR) | 20 (18 – 24) |
| ≥ 30/min, n (%) | 125 (11.0) |
| **Systolic blood pressure (mm Hg)** |  |
| median (IQR) | 132 (119 – 147) |
| < 90, n (%) | 28 (2.5) |
| **Temperature (°C)** |  |
| median (IQR) | 99 (98 – 101) |
| < 35 or ≥ 40, n (%) | 8 (0.7) |
| **Pulse (per minute)** |  |
| median (IQR) | 93 (82 – 106) |
| ≥ 125/minute, n (%) | 60 (5.3) |
| ***Laboratory Values*** |  |
| **Arterial pH** |  |
| median (IQR) | 7.45 (7.41 – 7.47) |
| < 7.35, n (%) | 15 (1.3) |
| **Blood urea nitrogen (mg/dl) (11 mmol/liter)** |  |
| median (IQR) | 16.5 (12.0 – 24.0) |
| ≥ 30, n (%) | 165 (14.5) |
| **Sodium (mmol/liter)** |  |
| median (IQR) | 137 (134 – 139) |
| < 130, n (%) | 47 (4.1) |
| **Glucose mg/dl (14 mmol/liter)** |  |
| median (IQR) | 124 (106 – 158) |
| ≥ 250, n (%) | 91 (8.0) |
| **Hematocrit (%)** |  |
| median (IQR) | 40.1 (36.7 – 43.4) |
| < 30, n (%) | 32 (2.8) |
| **Partial pressure of arterial oxygen (mm Hg)** |  |
| median (IQR) | 72.0 (62.7 – 85.0) |
| < 60, n (%) | 16 (1.4) |
| **Creatinine Clearance (mL/min)** |  |
| median (IQR) | 96 (63 – 135) |
| ≥ 50, n (%) | 957 (85.8) |
| **D-Dimer level (ng/mL), n (%)** |  |
| Normal (≤ 0.5) | 868 (76.3) |
| Moderate (0.51 – 1.0) | 104 (9.1) |
| High (> 1.0) | 166 (14.6) |
| **Absolute Lymphocyte Count (x10^9^/L)** | 0.90 (0.68 – 1.00) |

Abbreviations: BMI, body mass index; PSI, pneumonia severity index; SNF, skilled nursing facility; WHO OSS, World Health Organization Ordinal Scale Score; DNR, do not resuscitate; AMS, altered mental status; SD, standard deviation.
